# Antihistamine-Induced Hepatitis: 2 Cases Involving Loratidine

**DOI:** 10.1155/2016/6890313

**Published:** 2016-05-15

**Authors:** Hafiz Arshad, Arsalan Khan, Usama Assad, Muaiad Kittaneh, Charles Berkelhammer

**Affiliations:** University of Illinois, Oak Lawn, IL 60453, USA

## Abstract

Antihistamine-induced hepatitis is rare. We present 2 cases of antihistamine-induced hepatitis with autoimmune features, caused by loratidine. One case was confirmed by rechallenge. Identifying and discontinuing the offending agent are essential for treatment.

## 1. Introduction

Antihistamine-induced hepatitis is rare. We present 2 cases of antihistamine-induced hepatitis caused by loratidine. Both cases had autoimmune features. One case was confirmed by rechallenge.

## 2. Case Report

### 2.1. Case  1

A 49-year-old male with a past medical history of allergic rhinitis presented for evaluation of abnormal liver biochemistries. He also complained of lower extremity paresthesia. He had been taking loratidine 10 mg daily for the past year. He did not take other medications, alcohol, or herbs. His ALT was 675 U/L, AST was 305 U/L, and bilirubin was 2.9 mg/dL. His INR was 1.2. Extensive serological work-up was negative, including hepatitis A IgM, hepatitis B surface antigen, hepatitis B core antibody, hepatitis C antibody, hepatitis C RNA, cryoglobulins,* Cytomegalovirus* IgM, Epstein-Barr virus IgM, hepatitis E antibody, antinuclear antibody, anti-cytoplasmic antibody, liver kidney microsomal antibody, and soluble liver antigen. Quantitative immune globulins were normal. Ceruloplasmin and C3 and C4 were normal. Celiac antibodies and HLA DQ2 and DQ8 were negative, as was small bowel biopsy. Antismooth muscle antibody was positive at 1 : 40. Circulating immune complexes were positive at 45 ugE/mL (normal < 8 ugE/mL). A liver biopsy showed panacinar and portal hepatitis. There was lymphocytic infiltrate within portal tracts, sparing bile ducts. The hepatitis was grade 4/4 with zone necrosis but no fibrosis. EMG and sural nerve biopsy suggested that his lower extremity paresthesia was due to small fiber axonal sensory peripheral neuropathy. Loratidine was discontinued. Prednisone was initiated at 40 mg/day with rapid resolution of his hepatitis. The prednisone was gradually tapered over 9 months. He was diagnosed with loratidine-induced hepatitis with autoimmune features and small fiber peripheral neuropathy. His liver biochemistries remained normal for 10 years after discontinuation of loratidine.

### 2.2. Case  2

A 50-year-old male with past medical history of allergic rhinitis presented with jaundice and fatigue. He had been on loratidine 10 mg daily on an intermittent basis for a few years. His bilirubin was 9.1 mg/dL, ALT was 2,495 U/L and alkaline phosphatase was 189 U/L. Extensive serological work-up was negative including hepatitis A IgM, hepatitis B surface antigen, hepatitis B core antibody, hepatitis C antibody, hepatitis C RNA,* Cytomegalovirus* IgM, Epstein-Barr virus IgM, hepatitis E antibody, antinuclear antibody, anti-cytoplasmic antibody, anti-liver kidney microsomal antibody, and soluble liver antigen. Quantitative immune globulins were normal. Anti-F1 actin was positive at 1 : 31, and anti-mitochondrial antibody was positive at 1 : 88. Liver biopsy showed chronic hepatitis with lymphocytic infiltrate in the portal triads, as well as scattered eosinophils and rare groups of plasma cells. Trichrome stains revealed areas of portal fibrosis. Loratidine was discontinued, with subsequent normalization of his liver biochemistries. He restarted loratidine 10 mg daily. His liver biochemistries promptly increased to an ALT of 1,800 U/L, AST of 1,027 U/L, and a bilirubin of 3.9 mg/dL. Loratidine was discontinued. Prednisone 40 mg/day and ursodeoxycholic acid 15 mg/kg/day were initiated. His liver biochemistries rapidly normalized. Prednisone was gradually tapered over 5 months and discontinued. His liver biochemistries have remained normal for 1.5 years of follow-up.

## 3. Discussion

Antihistamine-induced hepatitis is rare. There have been a few reported cases causing both a hepatocellular [1–11] and a cholestatic [12, 13] picture. In the first patient we describe, loratidine-induced hepatitis was associated with immune features, specifically circulating immune complexes, low titer antismooth muscle antibody, lymphocytic infiltration on liver biopsy, and a rapid response to corticosteroids. In our second patient, loratidine-induced hepatitis was confirmed by rechallenge. In this later case, the patient was susceptible to autoimmunity, as evidenced by low titer elevation of anti-F1 actin and low titer anti-mitochondrial antibody. Immune features in this patient included lymphocytic infiltration on liver biopsy and rapid response to corticosteroids. This suggests that loratidine triggered an immune mediated injury resembling an overlap syndrome between autoimmune hepatitis and primary biliary cirrhosis.

Drug-induced liver injury may have autoimmune features, suggesting that an immune mechanism contributes to liver injury. In addition, drug-induced hepatitis may mimic classical autoimmune hepatitis, as can be seen with autoimmune hepatitis induced by minocycline, alpha methyldopa, nitrofurantoin, and anti-TNF blocking agents [14–16]. Covalent binding of a reactive metabolite to a hepatocyte surface protein, formation of a neoantigen, activation of CD8 T lymphocytes, and deficient immune regulatory systems are likely involved [15]. Discontinuing the offending drug is essential for treatment.

We speculate that loratidine incites an immune mediated hepatocellular injury and can trigger an autoimmune-like hepatitis in a genetically susceptible host.

## References

[1] Pompili M., Basso M., Grieco A., Vecchio F. M., Gasbarrini G., Rapaccini G. L. (2004). Recurrent acute hepatitis associated with use of cetirizine. *Annals of Pharmacotherapy*.

[2] Jurawan R., Smith A. (2010). Severe hepatitis in a primary sclerosing cholangitis patient receiving cetirizine therapy. *New Zealand Medical Journal*.

[3] Sánchez-Lombraña J. L., Álvarez R. P., Sáez L. R., Oliva N. P., Martínez R. M. (2002). Acute hepatitis associated with cetirizine intake. *Journal of Clinical Gastroenterology*.

[4] Watanabe M., Kohge N., Kaji T. (2001). Severe hepatitis in a patient taking cetirizine. *Annals of Internal Medicine*.

[5] Ekiz F., Yüksel I., Ekiz Ö., Coban S., Basar Ö., Yüksel O. (2011). Levocetirizine induced hepatotoxicity in a patient with chronic urticaria. *Annals of Hepatology*.

[6] Schiano T. D., Bellary S. V., Cassidy M. J., Thomas R. M., Black M. (1996). Subfulminant liver failure and severe hepatotoxicity caused by loratadine use. *Annals of Internal Medicine*.

[7] Schöttker B., Dösch A., Kraemer D. M. (2003). Severe hepatotoxicity after application of desloratadine and fluconazole. *Acta Haematologica*.

[8] Pérez R., Rodrigo L., Pérez R., de Francisco R. (2005). Acute cholestasis related to desloratidine. *World Journal of Gastroenterology*.

[9] Kew M. C., Segel J., Zoutendyk A. (1973). ‘Hypersensitivity hepatitis’ associated with administration of cyclizine. *British Medical Journal*.

[10] Bera F., Siproudhis J. P., Jonville-Bera A. P. (1993). Cytolytic hepatic involvement after administration of cetirizine (Zyrtec). *Gastroentérologie Clinique et Biologique*.

[11] Mason J., Reynolds R., Rao N. (1999). The systemic safety of fexofenadine HCl. *Clinical and Experimental Allergy*.

[12] Rodriguez-Gomez S. J., Zamora-Martinez T., Bailador-Andres C. (2009). Colestasis intrahepática grave asociada a la ingesta de cetiricina. *Gastroenterología y Hepatología*.

[13] Fong D. G., Angulo P., Burgart L. J., Lindor K. D. (2002). Cetirizine-induce cholestasis. *Journal of Clinical Gastroenterology*.

[14] Bhat G., Jordan J., Sokalsi S. (1998). Minocycline-induced hepatitis with autoimmune features and neutropenia. *Journal of Clinical Gastroenterology*.

[15] Czaja A. J. (2011). Drug-induced autoimmune-like hepatitis. *Digestive Diseases and Sciences*.

[16] Efe C. (2013). Drug induced autoimmune hepatitis and TNF-*α* blocking agents: is there a real relationship?. *Autoimmunity Reviews*.

